# Policy implications for familial searching

**DOI:** 10.1186/2041-2223-2-22

**Published:** 2011-11-01

**Authors:** Joyce Kim, Danny Mammo, Marni B Siegel, Sara H Katsanis

**Affiliations:** 1 Center for Genome Ethics, Law & Policy, Duke Institute for Genome Sciences & Policy, Duke University, Box 90141, Durham, NC 27708, USA

**Keywords:** genetic identity, forensic genetics, DNA typing, DNA data bank, likelihood functions

## Abstract

In the United States, several states have made policy decisions regarding whether and how to use familial searching of the Combined DNA Index System (CODIS) database in criminal investigations. Familial searching pushes DNA typing beyond merely identifying individuals to detecting genetic relatedness, an application previously reserved for missing persons identifications and custody battles. The intentional search of CODIS for partial matches to an item of evidence offers law enforcement agencies a powerful tool for developing investigative leads, apprehending criminals, revitalizing cold cases and exonerating wrongfully convicted individuals. As familial searching involves a range of logistical, social, ethical and legal considerations, states are now grappling with policy options for implementing familial searching to balance crime fighting with its potential impact on society. When developing policies for familial searching, legislators should take into account the impact of familial searching on select populations and the need to minimize personal intrusion on relatives of individuals in the DNA database. This review describes the approaches used to narrow a suspect pool from a partial match search of CODIS and summarizes the economic, ethical, logistical and political challenges of implementing familial searching. We examine particular US state policies and the policy options adopted to address these issues. The aim of this review is to provide objective background information on the controversial approach of familial searching to inform policy decisions in this area. Herein we highlight key policy options and recommendations regarding effective utilization of familial searching that minimize harm to and afford maximum protection of US citizens.

## Introduction

The Combined DNA Index System (CODIS) database was established by the Federal Bureau of Investigation (FBI) to aid in the exchange of DNA profiles in forensics investigations by facilitating data sharing and comparisons of short tandem repeat (STR) profiles at the local, state and national levels. The DNA Identification Act of 1994, a subtitle of the Violent Crime Control and Law Enforcement Act of 1994, authorizes the Director of the FBI to establish "an index of DNA identification records of persons convicted of crimes, and analyses of DNA samples recovered from crime scenes and from unidentified human remains" [1]. The CODIS database software facilitates DNA data sharing and comparisons of STR profiles across the local, state and national levels [2].

CODIS was originally intended to be utilized for the investigation of violent crimes and sex offenses by using exact matches between DNA evidence and DNA database profiles to develop new investigative leads and to aid in missing persons identifications [2]. States have set their own standards and regulations governing when an individual shall be profiled for inclusion in the database. States also set their own policies and schedules regarding the data entry and search schedules. Since 1994, CODIS has been expanded in many states to include profiles of nonviolent offenders and arrestees and has become an invaluable tool for law enforcement in a variety of investigative contexts [3].

Recently, law enforcement agencies have successfully used existing CODIS software to detect partial matches through a low-stringency search, which allows for mismatches at some markers, in addition to absences and dropouts allowed in moderate-stringency searches. Investigators commonly use a low-stringency search of CODIS when facing an evidence sample developed from multiple sources or if the DNA evidence is of low quality [2]. Low-stringency searches also can identify biological relatives by revealing genetic similarities between individuals, which led to a nationwide call to use CODIS for intentional familial searches [4]. In crime investigations, "familial searching" is defined as the intentional search of an offender DNA database for inexact matches between DNA evidence profiles and offender and arrestee DNA profiles. Upon the identification of one or more partial match profiles, law enforcement may investigate purported family members of the partial matches as suspects. Such familial searching has identified and convicted suspects, allowed investigators to reopen cold cases and exonerated wrongfully convicted individuals [5]. Additionally, familial searching has led to three successful convictions in California and Colorado and has resulted in leads in many more ongoing cases [6].

In March 2008, the FBI determined that familial searching policies should be decided by individual states [7]. As of June 2011, California, Colorado, Texas and Virginia had passed state legislation permitting familial searching, and Maryland and Washington, DC, had banned familial searching (see Table 1) [8-13]. Despite individual states' discretion, there is an expectation of reasonable uniformity among states regarding the use of CODIS [2]. To address this expectation, at least in part, policymakers are considering a national platform that would allow crime investigators to run familial searches of CODIS. In July 2010, federal legislation (H.R.6011) was introduced in the US House of Representatives to authorize the FBI to conduct familial searches primarily in cold case investigations of violent or sex crimes [14].

**Table 1:** State policies for familial searching and partial match disclosure

	States with formal familial searching policies			
	
Parameter	California	Colorado	Texas	Virginia
Proportionality				
Violent crimes	X	X	X	X
Nonviolent crimes		X		
CODIS offenders searched				
Convicted offenders	X	X	X	X
Arrestees		X	X	X
Tools for narrowing suspect pool				
IBS	X	X	X	X
Likelihood ratio	X	X	X	X
YSTR analysis	X	X	X	X
Other policy specifics				
Requires profiles on all 13 CODIS loci	X	X	X	X
Requires evidence profile to be single source	X		X	
Permits mixtures with clearly defined profiles		X		X
Oversight committee	X^a^		X^b^	
Requires specialized training of law enforcement		X		
Requires public record verification before follow-up		X		
States permitting partial match disclosure [4]				
Arizona, Connecticut^c^, Florida, Missouri and Nebraska^d^, Nevada, New York, Oregon and Washington State^e^, Wyoming				

CODIS = Combined DNA Index System; IBS = identity-by-state; YSTR = Y-chromosome short tandem repeat. ^a^Familial Search Committee. ^b^Committee of four CODIS analysts recommends when to conduct familial searches. ^c^Restricted to profiles where a genetic similarity "must raise the hair on the back of the analyst's neck to be worth pursuing" (Ram (2011) [4], p 770). ^d^"Targeted analysis" may be conducted on a case-by-case basis and upon specific request (Ram (2011) [4], p 772). ^e^Policy addresses partial matches derived from routine moderate-stringency searches.

Familial searching has immense potential to help law enforcement develop leads in investigations that would otherwise go unsolved. This novel approach presents both technical and logistical challenges for law enforcement, however, and raises distinct ethical, social and legal concerns. As policies governing familial searching develop, policymakers, law enforcement agencies and the public may consider options for improving the effectiveness and reliability of familial searches, the potential effects on society and families, the associated Fourth Amendment implications, and the costs and benefits to law enforcement. In this review, we examine the challenges presented by familial searching and the policies adopted by some states to address these challenges.

### Narrowing the suspect pool

For a familial search, investigators run a low-stringency search with the intention of identifying a relative of the perpetrator. Such a low-stringency search is most likely to result in parent-offspring relationships, whereas sibling and avuncular relationships (for example, cousins and half-siblings) are less likely to result. A familial search typically results in a list of multiple candidate partial matches whose family members may eventually be investigated further as suspects or persons of interest.

A perpetrator and a profiled offender are most likely to share genetic similarities if they are close biological relatives, but partial matches might also include random unrelated individuals profiled in CODIS whose DNA profiles are by chance similar to the evidence sample profile. Consequently, when a familial search is performed, the resulting suspect pool can contain from a few up to hundreds or more suspects, depending on the search parameters used, making the initial pool of CODIS partial matches and their biological relatives too large for a manageable investigation [15].

Additional analysis is necessary to verify relatedness between the evidence profile and the partially matched offender [3,15]. Taking measures to improve the reliability of familial searching will maximize the efficiency of investigations and limit unwarranted scrutiny on innocent individuals. Several states have also adopted approaches to narrow a suspect pool to a single or a few candidate persons of interest.

Since the DNA Identification Act of 1994 does not explicitly authorize familial searches at the federal level, searches of the National DNA Index System (NDIS) are not currently conducted [1,16]. Because moderate- and low-stringency CODIS searches were originally intended to match DNA profiles from evidence samples directly to CODIS profiles, the search results do not statistically rank partial matches or take into consideration allele frequency. Likewise, the latest edition of the CODIS database (CODIS 7.0) will not include familial searching capabilities [17]. However, in March 2008, the Scientific Working Group on DNA Analysis Methods (SWGDAM) proposed protocols for pursuing incidentally found partial matches and made recommendations on further analysis to confirm relatedness [7].

#### Retesting Y-chromosome short tandem repeat markers

Retesting of evidence and candidate profiles for Y-chromosome STR (YSTR) markers can greatly reduce the number of coincidental matches in the suspect pool. Because profiles in CODIS are predominantly males, paternal relatedness between two profiles may often be verified by YSTR analysis [3]. SWGDAM recommended that YSTR typing be conducted on candidates identified through a familial search of the DNA evidence [7,18]. In California, YSTR typing of DNA evidence is completed prior to the familial search request [9]. California, Colorado and Texas policies require YSTR analysis of male candidate results to be conducted before identifying information on the partial match profile is released to investigators [9,11,12].

#### Mitochondrial DNA

Analysis of mitochondrial DNA (mtDNA) serves a similar function to YSTR analysis in reducing coincidental partial matches and narrowing the pool of true relatives by implicating maternal lineage [19,20]. However, sequencing of mtDNA presents a greater technical challenge and expense than YSTR analysis, so routine mtDNA testing may be impractical [21].

#### Statistical analysis: identity by state

The identity-by-state (IBS) statistical approach infers genetic similarity based on the number of matching markers between two profiles, regardless of how individual markers are inherited. When using the IBS approach, analysts rank matches based on the highest number of matched markers to the lowest number, with a full match being 26 shared alleles [18,20]. For familial searching, this approach can prioritize an entire data set of potential relatives without complicated statistical analysis. However, the IBS approach does not take into account allele frequency, population size and other factors that affect the likelihood that two samples are genetically related [18]. For instance, parent-child relationships are easily detected when two profiles share at least one allele at each marker; however, the IBS between a parent and child could be as low as 13 of 26 shared alleles and thus could be falsely excluded from a candidate partial match list [20]. In California, software for familial searching generates partial matches using the IBS strategy, limiting the pool to candidate profiles sharing 15 or more (of 26 possible) alleles with the evidence profile, which must be a single-source profile [9]. This IBS target will likely be adjusted as the database expands and as the software develops (M Chamberlain, Deputy Attorney General, California Department of Justice, personal communication).

#### Statistical analysis: likelihood ratio

In familial searching, a likelihood ratio (or kinship index) compares the probability of two profiles' being from related sources to the probability of the two profiles' being unrelated. Calculating a likelihood ratio takes into account all 13 markers typed in a CODIS profile, the allele frequencies of the 26 detected alleles and potential mutation events at each marker. The likelihood ratio can vary based on the frequency of each marker across the population, the number of loci compared in the profiles and the prior odds of the alleged relationship [19]. The likelihood ratio calculation allows investigators to rank the individuals within the pool of candidates according to the probability that the evidence profile is related to the CODIS profile [3,18,20]. In 2008, SWGDAM recommended that a threshold likelihood ratio be used to calculate kinship [7]. To carry out Colorado's familial search policy, the Denver District Attorney's office and the Denver Police Crime Lab designed software that combines IBS and likelihood ratios for use in familial searches [11]. Virginia's authorities adopted the software developed in Colorado to conduct familial searches in Virginia [10]. In Texas, familial searches are conducted using software that was developed by the University of North Texas, which uses a combination of IBS and likelihood ratio and includes YSTR analyses [12]. California uses software to generate likelihood ratios for all profiles from the IBS screen (sharing 15 or more alleles). The software returns profiles above an established likelihood ratio threshold and can be used to rank candidate profiles by statistical relatedness [9].

#### Retesting with additional markers with a focus on rare alleles

To improve the reliability of hits from familial searches, investigators can type markers in addition to the standard 13 STRs [18]. Additional markers could improve the precision of inferences of familial relatedness and might also unveil rare alleles. All STR alleles vary, with different frequencies occurring among different populations. Close relatives are more likely than two random individuals to share rare alleles. If an evidence profile happens to exhibit a rare allele at one of the thirteen STRs, investigators may choose to first focus the investigation on partial match candidates who also carry the rare allele.

#### Public record review

Traditional police investigation of public records may support or refute suspected biological relationships in a candidate partial match list. Such public records review is costly but can reduce concerns about unwarranted investigation of families unrelated to the perpetrator of the crime [11]. In Colorado, crime investigators are expected to attempt to verify familial relations through background checks of the partial match individual and family members using public resources, such as court and jail records, criminal history checks, investigative reports and vital records [11].

### Ethical, legal and social issues

Familial searching approaches can either positively or negatively affect individuals, families, certain racial groups and society in general, inevitably raising ethical and privacy questions. Proponents of familial searching assert that the potential to improve public safety and ensure justice outweigh any costs to individual and family privacy, especially if mechanisms are in place to minimize harm [5,22,23].

Between 2003 and 2010, the United Kingdom successfully used familial searching to obtain convictions in 19 cases [24]. To address ethical challenges, the United Kingdom established the National DNA Database Ethics Group, which monitors all ethical and human rights concerns with respect to searches [25]. This ethics group covers a wide range of topics, including familial searching as well as general privacy and ethical issues associated with the use and storage of DNA, and makes policy recommendations, such as whether to implement routine Y-chromosome testing [26]. Similarly, California formed a Familial Search Committee (FSC) comprised of law enforcement officials, attorneys and scientists within the California Department of Justice, which reviews familial search requests submitted by law enforcement (M Chamberlain, Deputy Attorney General, California Department of Justice, personal communication). The members review the progress of cases and provide legal and ethical checkpoints at major steps in the investigation. The California FSC requires law enforcement officials to sign a Memorandum of Understanding stating that they will follow through with their request and with the conditions of approval of familial searching [9].

#### Family and personal privacy

Opponents of familial searching warn that, as a by-product of familial searches in an investigation, family members may learn information about relatives that was previously unknown to them [27]. For example, family members may not be aware that their relative was arrested or convicted of a crime. Familial search investigations may also reveal a genetic relationship previously unknown to the individuals [27,28]. The revelation of such facts could have a profound emotional impact on entire families, potentially leading to domestic violence or estrangement [5]. To mitigate the potential for harm precipitated by such incidental findings, familial searching policies may impose verification of familial relationships through the public records as a prerequisite for questioning the suspects uncovered in the familial search, such as the requirement contained in Colorado's familial search policy [11]. Advocates have noted that familial searching minimally affects an individual's sense of privacy and liberty because individuals investigated as a result of a familial search remain unaware that they are under police scrutiny until law enforcement officials identify further cause for interrogation [22,23].

#### Abuse of power

Because familial searching is intended to develop a suspect pool when conventional investigation of available evidence has not created leads, it by design opens the investigation of potentially innocent individuals based on their genetic material [29]. Some argue that familial searching carries a risk for increased genetic surveillance in that law enforcement officials may investigate individuals primarily on the basis of their genetic information [3,30,31]. Concerns about genetic surveillance are exacerbated by the additional risk of abuse of power by law enforcement officers and agencies [27]. Although most policies permit familial searching only after all other investigative options have been exhausted, law enforcement officials may use familial searching as a routine part of their investigations. Routine familial searching may aggravate public perceptions that law enforcement exploits CODIS to troll for suspects and invade individuals' privacy [32]. To date no such reports of abuse have surfaced. Conversely, some people believe it is their duty as citizens to cooperate and assist with law enforcement, and proposed tools to solve crime typically garner public support. To limit the abuse of power, California, Texas and Virginia authorize familial searches only after investigators have exhausted all other leads [9,10,12]. The Colorado policy allows the Colorado Bureau of Investigation to conduct routine familial searches and permits crime investigators to follow up on potential matches obtained from a search [11].

#### Constitutionality

Legal scholars have taken various stances on the constitutionality of familial searches. Their opinions are based on two core questions about when a search occurs and whether a search is reasonable. The Fourth Amendment provides protections against unreasonable searches and seizures [33]. US courts continue to debate the Fourth Amendment implications of using a DNA sample for purposes beyond the initial creation of a profile [32,34,35]. The collection of physical biological material for forensic analysis has been termed a search under the Fourth Amendment [36]. In familial searching, scholars have posited that the timing of the search affects who may be considered the subject of a Fourth Amendment search. Consideration of who is subject to a search may shape how courts and legal experts assess the privacy considerations implicated by the search and whether law enforcement officials may conduct a reasonable search based on the progress of an investigation. In some jurisdictions, a search warrant issued on the basis of a showing of probable cause is required to conduct a reasonable search. In others, the justification for a search can be established with reasonable suspicion or suspicionless cause (L Dame, personal communication, class lecture entitled "Familial searches of DNA databases and the Fourth Amendment," Spring 2011, Duke Genome Sciences and Policy Capstone course, Duke University). In some instances, suspicionless searches are allowed on a case-by-case basis, with courts weighing individual privacy rights against the government's interest in protecting the public from crime. To ensure that a search is reasonable, courts may apply the balancing test to weigh individuals' rights protecting them from unreasonable search and seizure against the government's duty to preserve public safety.

Some scholars posit that, because biological materials are not collected directly from relatives under investigation during a familial search, the legal search occurs when the low-stringency DNA database search is run, such that investigators effectively include relatives of convicted offenders and arrestees in a database search. From this perspective, in a familial search, CODIS offenders' biological relatives may be considered the individuals being searched and thus are protected under the Fourth Amendment. Others argue that the legal search occurs during the creation of the profile: that is, when the sample is collected, tested or entered into the database (L Dame, personal communication, class lecture entitled "Familial searches of DNA databases and the Fourth Amendment," Spring 2011, Duke Genome Sciences and Policy Capstone course, Duke University). From this perspective, as long as law enforcement officials properly obtain a genetic sample during a search of an offender, subsequent testing and analysis of the DNA may not be protected by the Fourth Amendment [37].

Because investigators typically use familial searching when existing leads do not point to a specific suspect, a familial search may require justification for suspicionless cause. Some scholars propose that only crimes that pose a substantial threat to public safety warrant the use of familial searching [3,5]. California, Texas and Virginia choose to restrict familial searching only for violent or sexual crimes because the heinous nature of these crimes compels a strong response from law enforcement officials [9,10,12]. On the other hand, because many criminals commit minor crimes before they engage in major violent crimes, the use of familial searching to investigate lesser crimes could be justified to prevent future felonies. Colorado does not limit familial searching to violent crimes, but requires that crime investigators submit written requests to conduct familial searches when the crime under investigation poses a substantial public safety concern and conventional investigative approaches have been exhausted [11]. Colorado also authorizes nontestimonial identification orders, allowing law enforcement agencies to obtain DNA samples based on suspicion lower than probable cause. Courts may issue nontestimonial identification orders for the collection of various specimens, including fingerprints, blood, urine and hair [38]. Colorado policymakers may extend nontestimonial identification orders to allow law enforcement to conduct familial searches in cases without individualized suspicion.

While convicted felons may be profiled and searched nationwide, state policies differ with regard to whether arrestees and juveniles may be profiled, whether they continue to have a reduced expectation of privacy after their release from incarceration, or whether they can be profiled after their records are expunged. Some argue that arrested individuals and juveniles may reasonably expect to have decreased privacy during the time of arrest and therefore may be included in DNA databases. Others posit that arrestees' presumed innocence and juveniles' minority status might necessitate greater privacy protections than those granted to convicted felons [22,36]. The ongoing debates about the creation and retention of arrestee and juvenile profiles may extend to familial searching. California's State DNA Index System (SDIS) separates the arrestee index from felons' profiles, so familial searches of California's SDIS exclude arrestees [9]. Virginia, Texas and Colorado, on the other hand, include both offenders and arrestees in their SDIS familial searches [10-12].

Because familial searching has not yet been challenged in the courts, how US courts will assess the Fourth Amendment implications of familial searching is unknown [31,34,35]. Legal experts will continue to debate the permissibility of familial searching under the Fourth Amendment, but the parameters for allowing familial searching will remain uncertain until the Supreme Court addresses this issue.

#### Disparities in select populations

Familial searching has the potential to exacerbate the racial bias already documented in the criminal justice system [3,39,40]. Because of their disproportionately high rates of arrests, prosecutions and convictions, members of racial minority groups are significantly overrepresented in CODIS. With the use of familial searching, members of racial groups are searched in proportion to their relative representation in the US justice system. It has been estimated that familial searching could survey up to 17% of the total African American population as opposed to only 4% of the Caucasian population [37]. Despite the potential for disproportionate scrutiny of racial minorities in familial searching, victims of violent crimes are often members of racial minority populations, such that improved conviction rates achieved with the use of familial searching also benefit those communities [41].

Maryland banned familial searching of the state-specific database as part of legislation to expand its DNA databases to include arrestees of violent crimes because of the disproportionate number of racial minorities subject to arrest [17]. In other words, legislators feared that allowing familial searching of an arrestee database could disproportionately focus law enforcement efforts on a large group of people who are primarily defined by their race, despite their never having committed a crime (S Mercer, personal communication, class interview with key informant on the Maryland decision to ban familial searching, Spring 2011, Duke Genome Sciences and Policy Capstone course, Duke University).

State-by-state familial searching policies may also result in a disparate focus by law enforcement officials on families of low socioeconomic status. Investigations involving familial searching presently are conducted within a handful of states. Consequently, biological relatives who live outside the state in which the crime was committed will not be subject to searches of the state-specific database. Because families of low socioeconomic status tend to have decreased mobility, they are more likely to live within the same state, city and/or town as their relatives [42]. As a result of state-by-state familial searching policies, families of low socioeconomic status are searched and investigated more robustly than families of high socioeconomic status.

#### Economic impact on laboratories and law enforcement agencies

Data are needed to understand the actual impact of familial searching on all facets of crime investigation. Familial searching may burden crime laboratories, crime investigators and the justice system in terms of cost and time. Because familial searching will increase the number of suspects identified on the basis of DNA forensics, law enforcement agencies may be burdened with an increased workload, particularly when they are required to investigate a broad suspect pool. Once a list of partial matches is made, law enforcement must decide whether to expend resources to retest samples for additional markers and follow up on multiple leads.

Familial searching requires that evidence be retested for additional markers and to review the results of a search, thus increasing the workload of crime laboratories. Existing crime laboratory backlogs may worsen with increased searching and retesting demands. Because many crime laboratories prioritize high-profile and violent crimes, familial search requests for violent crimes may jump ahead of other backlogged DNA-based cases [43].

#### Logistics and CODIS authority

The authority of the FBI to alter CODIS software is limited by the DNA Identification Act of 1994 [1]. This federal law restricts the purpose of CODIS to the identification of suspects as opposed to the development of investigative leads, presenting a potential hurdle to be cleared before a national familial search platform can be developed. Because familial searches are conducted at the state level, challenges in coordinating data and communicating across jurisdictions persist. It remains unclear whether CODIS software can be modified or adapted to more efficiently yield leads or whether authority must be granted by federal legislation.

### Policy options

In implementing a familial search policy, there are several options for determining when and how the approach is used, the scope of a search and how to narrow the suspect pool to eliminate coincidental genetic matches. The six policy options outlined below might be considered in an effort to (1) maximize the efficiency of a system, (2) limit the burden on laboratories and law enforcement, (3) address some of the aforementioned impacts on society and families and (4) balance ethical implications and individual rights with the duty of law enforcement officials and agencies to carry out their responsibilities and protect the public's safety.

#### 1. The FBI could be granted authorization to develop software to search CODIS for biological relatives

CODIS software is not designed to perform familial searches effectively, yet a national platform is the best approach to ensure consistency across jurisdictions. Consistent application of familial searching will require standard tools to manage the pools of candidate suspects and minimize both the burden on law enforcement officials to investigate leads and the privacy intrusion on families. Software designed to conduct familial searches could incorporate both IBS and likelihood ratio analyses to maximize efficiency. FBI officials have indicated that they currently do not have the authority to make such changes [17].

#### 2. National policy could be established to type YSTR markers for all prospective CODIS profiles

Routine analysis of YSTR markers when a CODIS profile is initially typed would improve the efficiency of familial searching by removing the need for retesting, hence eliminating one of the analytical burdens on laboratories. Individual states could develop policies for retesting stored DNA samples for YSTR markers based on their available resources and familial searching needs.

#### 3. A national advisory consortium could be established to guide the development of statistical tools for familial searching

Scientists and policymakers are evaluating which statistical methods and analytical tools will efficiently limit the pool of suspects without excluding the actual perpetrator. As policies and technologies develop and as the CODIS database expands, the statistical parameters for a search will need to be reviewed and adjusted. Advice from experts in relationship testing, statistics and population genetics will be crucial for consistent application of familial searching among the various population pools across the United States. An advisory consortium modeled on the DNA Advisory Board could guide the establishment and revision of the statistical criteria used to develop familial searching policies, both nationally and state-by-state. Statistical tools, including the IBS approach and likelihood ratios, can be used to rank partial matches to optimize the likelihood that a match corresponds to a true biological relationship. However, an advisory consortium may have limited authority to enforce or regulate policy.

#### 4. A national advisory consortium could be established to review cases and serve an ethics advisory function in policy implementation

Familial searching has raised ethical concerns because of its potential to profile certain socioeconomic and minority groups disproportionately, alter family dynamics and diminish individuals' sense of liberty. The development of an ethics oversight board is one option for ensuring that the social and ethical implications of familial searching are addressed and incorporated into policy and decision-making. Federal policy-making bodies could model an ethics advisory board upon California's Familial Search Committee and the UK Ethics Group.

A national advisory consortium would provide guidance on the ethical boundaries and privacy concerns of familial searching for states considering legislation and investigating ongoing cases. A national resource would provide a broad perspective on nationwide trends and emerging issues related to US Constitutional rights. Additionally, an advisory consortium might address privacy concerns by making recommendations regarding police investigation approaches. Although policy could ultimately be left to the discretion of individual law enforcement offices, a national consortium could (a) recommend safeguards for when partial match individuals' identities may be disclosed to law enforcement officials for further investigation or (b) advise law enforcement regarding the kind of information that may be disclosed to family members. An ethics advisory consortium has limited authority to enforce or regulate policy. However, a national ethics advisory consortium could provide guidance for review of familial search policies or investigations.

#### 5. States could determine for which crimes familial searching is appropriate

States may individually apply the balancing test to determine whether law enforcement should use familial searching routinely or on a case-by-case basis. Policy makers may assess the circumstances for which to conduct a familial search by weighing the needs to (a) prevent crime and protect the public interest and public safety and (b) protect against the infringement of individual privacy rights, avoid social ramifications (including disproportionate profiling of racial minority populations), ameliorate impacts on families and limit the potential financial and time burdens of familial searching on individual crime laboratories and police departments. Additionally, by using familial searching only for violent crimes, for example, law enforcement officials could reduce unnecessary costs and save time while aiding investigations of crimes that pose the most critical threats to public safety.

#### 6. States could determine whose DNA profiles can be used to conduct a familial search

In addition to deciding when a familial search is appropriate, concerns also arise over whose DNA profiles can be used for a familial search. Inclusion in a DNA database is no longer limited to individuals convicted of violent crimes. Some critics of familial searching argue that running familial searches of databases that include arrestees may by default include searches of innocent individuals whose privacy rights have not been diminished (as a convicted offenders' rights have been) [44,45]. Individual state policies may set different parameters for familial searches by narrowing or widening them on the basis of categories of offenders. Such searches may exclude arrestees or juveniles, thus limiting the familial search to adult individuals with a documented diminished expectation of privacy. Conversely, the search may include these categories to provide the broadest scope.

## Conclusion

Familial searching offers law enforcement a powerful tool for apprehending criminals, revitalizing cold cases and exonerating wrongfully convicted individuals. Development of familial searching policies should take into account open questions regarding the impact of familial searching policies on select populations and how to minimize personal intrusion on relatives of individuals in the DNA database. As policies develop and jurisdictions establish procedures, we will better understand the societal impact of familial searching.

## Abbreviations

CODIS: Combined DNA Index System; FBI: Federal Bureau of Investigation; FSC: Familial Search Committee; IBS: identity by state; mtDNA: mitochondrial DNA; NDIS: National DNA Index System; SDIS: State DNA Index System; STR: short tandem repeat; YSTR: Y-chromosome short tandem repeat.

## Competing interests

The authors declare that they have no competing interests.

## Authors' contributions

JK, DM and MS each independently researched aspects of the review topic under the guidance and advisement of SHK. JK and SHK wrote the manuscript. All authors read and approved the manuscript.
